# The Influence of Treated and Untreated Subclinical Hypothyroidism on Metabolic Profile in Women with Polycystic Ovary Syndrome

**DOI:** 10.1155/2021/8427150

**Published:** 2021-11-29

**Authors:** Karolina Kowalczyk, Patrycja Radosz, Kamil Barański, Dagmara Pluta, Dariusz Kowalczyk, Grzegorz Franik, Paweł Madej

**Affiliations:** ^1^Department of Endocrinological Gynaecology, Faculty of Medicine in Katowice, Medical University of Silesia, Katowice 40-752, Poland; ^2^Department of Epidemiology, Faculty of Medicine in Katowice, Medical University of Silesia, Katowice 40-752, Poland; ^3^Department of Anatomy, School of Medicine in Opole, University of Opole, Opole 45-052, Poland

## Abstract

**Background:**

Polycystic ovary syndrome (PCOS) and hypothyroidism are the most common endocrinological disorders among women of reproductive age. Since hypothyroidism occurs more frequently in PCOS patients, it is vital to explain its clinical impact.

**Aim:**

To evaluate the impact of subclinical hypothyroidism (SCH) and its treatment on the metabolic profile of patients with PCOS.

**Methods:**

190 women with PCOS phenotype A were enrolled in the case-control study. They were divided into three groups: 38 women with PCOS and subclinical hypothyroidism, 76 women with PCOS and SCH under thyroid replacement therapy, and 76 women with PCOS and normal thyroid function (control group). Serum lipids, fasting glucose, and insulin, as well as oral glucose tolerance tests were performed.

**Results:**

In the analyzed parameters, there were no statistic differences between the groups in relation to thyroid function. BMI turned out to be the main factor dividing the patients in terms of serum lipids, fasting glucose, fasting insulin, and oral glucose tolerance test. TSH was associated with total cholesterol and LDL cholesterol levels, whereas BMI has a dominant impact on HDL cholesterol, triglycerides, glucose, and insulin resistance.

**Conclusions:**

SCH is associated with mild lipid serum alterations in women with PCOS, but it is BMI to have a dominant impact on glucose and insulin. It seems that treatment of SCH in PCOS does not significantly alter lipid and glucose metabolism.

## 1. Introduction

Polycystic ovary syndrome (PCOS) is a disease that affects all stages of a woman's life. The full clinical picture of ovulation disorders, hyperandrogenism, and polycystic morphology of the ovaries in ultrasound is revealed in women of reproductive age [1, 2]. The cascade of events related to the coexistence of PCOS includes intrauterine growth restriction (IUGR), low birth weight, obesity, metabolic syndrome, cardiovascular diseases, noninsulin-dependent diabetes mellitus, and endometrial cancer. Insulin resistance and hyperinsulinemia seem to play a key role in the development of consecutive stages [3, 4]. A high percentage of insulin resistance in relation to the general population is observed regardless of BMI (Body Mass Index). At its root in PCOS there is a postreceptor defect in the early stage of intracellular insulin signaling. It consists in hyperphosphorylation of serine residues (and consequently inhibition) of the IRS-1 protein (IRS-1-insulin receptor substrate 1), which is bound to the insulin receptor and initiates the intracellular reactions characteristic for insulin [4]. These disorders are observed in peripheral tissues - adipocytes and skeletal muscles, but also in the cells of the ovarian granulosa and theca cell layers and in fibroblasts [4]. Around 70% of patients with PCOS show lipid metabolism disorders manifested by quantitative and qualitative changes in the parameters of the lipid profile and lipoproteins [5].

In recent years, there have been reports of a more frequent occurrence of hypothyroidism among patients diagnosed with PCOS-11–14% vs 1–2% in the control group [6–11]. In this age group, it is the most common subclinical form of hypothyroidism (SCH) [12, 13]. Possible causes include genetic, hormonal, and immunological factors [14]. It seems that obesity and insulin resistance may be the factors that feature the state of mild thyroid hormone deficiency and enhance the mutual adverse effects on metabolism [8].

Thyroid hormones are involved in the regulation of glycaemia at the central and peripheral levels [15]. At the central level, triiodothyronine (T3) modulates hepatic synthesis of glucose via the sympathetic nervous system. Peripherally, they enhance the expression of genes of the proteins which are responsible for glucose transport in skeletal muscles: the glucose transporter type 4 (GLUT-4) and proteins of the signaling cascade, which affects the increased basal and insulin-dependent transmembrane transport of glucose [15]. Thyroid hormones are also an important regulator of lipid levels in the organism by impacting their de novo synthesis, metabolism, and their degradation by modulating the expression of appropriate genes [16]. The metabolic changes observed in hypothyroidism and PCOS include insulin resistance, dyslipidemia, as well as excess weight and obesity [1, 17].

Therefore, it would seem that the coexistence of PCOS and hypothyroidism would correlate with more marked metabolic and hormonal changes. Mueller et al. described a significant dependence between the TSH value (as the most sensitive indicator of thyroid function) and insulin resistance, regardless of the age and BMI of the patients [18]. Reports from subsequent studies analyzing the parameters of carbohydrate management and lipid metabolism as well as phenotypic features of patients with PCOS and SCH revealed divergent results [19–21]. Finally, two meta-analyses from 2017 and 2018 investigated the impact of SCH on alterations of metabolic profile in PCOS, including total cholesterol, triglycerides, high-density lipoprotein (HDL), low-density lipoprotein (LDL), fasting gluocse and homeostatic model assessment of insulin resistance (HOMA-IR) [22, 23]. It was found that SCH causes small deviations, which do not appear to be of significant clinical importance in a short follow-up period. The authors admit that due to heterogeneity among studies (a cut-off for upper normal limi for TSH ranged from 2.5 to 5.0 mIU/L), some results should be treated with caution [22, 23]. PCOS phenotype was not assessed in the enrolled studies. The differences in fatty acids profile, as well as steroid pathways with relation to inflammatory state between androgenic and non-androgenic PCOS phenotype was underlined by Szczuko et al. [24, 25].

This study aimed to evaluate the lipid profile, glucose and insulin levels as well as HOMA-IR index in patients with PCOS and untreated SCH, and with PCOS and SCH under levothyroxine treatment, in relation to patients with PCOS and normal thyroid function.

## 2. Materials and Methods

### 2.1. Patients

190 women with PCOS phenotype A, aged 18–35, hospitalized at the Department of Gynecological Endocrinology of the Medical University of Silesia in Katowice from January 2014 to December 2018, were included in the study. The exclusion criteria were overt hypothyroidism, hyperthyroidism, previous thyroid surgery, hyperprolactinemia, congenital adrenal hyperplasia, Cushing's syndrome, diabetes, oral contraceptives, steroids, or hypoglycemic drug intake. The study group included 38 women with PCOS and untreated SCH, 76 women with PCOS and hypothyroidism under levothyroxine treatment, and 76 women with PCOS and normal thyroid function (control group). The subgroup analysis was performed after dividing our patients into two groups: normal weight (BMI <25 kg/m^2^) and overweight/obese women (BMI ≥25 kg/m^2^). The Ethics Committee of Medical University of Silesia approved of the use of retrospective data from the patients' files, no. KNW/0022/KB/325/18/19. All patients gave written informed consent, and their confidentiality and anonymity were protected.

### 2.2. Methods

Diagnosis of PCOS was based on the presence of all three Rotterdam Criteria: irregular menstrual cycles or amenorrhea, clinical and/or biochemical hyperandrogenism, and ultrasound assessment of polycystic ovaries (≥12 antral follicles in one ovary or ovarian volume ≥10 ml) [26]. Clinical hyperandrogenism was defined as the presence of hirsutism (≥8 points in the modified Ferriman-Gallwey score), acne, or alopecia. Biochemical hyperandrogenism referred to serum total testosterone >0.481 ng/mL, free testosterone >4.1 pg/mL, and/or free androgen index >5%. Gynecological ultrasound was performed with the use of machines Voluson 730 Expert and Voluson E8 Expert (GE Healthcare, New York, USA).

SCH was diagnosed based on TSH concentration above 4.2 mIU/L, with the normal level of free thyroxine-FT4 (9.01–21.88 pmol/L). In patients with PCOS and hypothyroidism treated with levothyroxine, thyroid dysfunction was diagnosed in the Endocrinology Outpatient Clinic. Oral levothyroxine therapy, in the form of medications containing levothyroxine sodium salt: Euthyrox N (Merck KGaA, Darmstadt, Germany) or Letrox (Berlin-Chemie AG, Berlin, Germany), was started at least 3 months before hospitalization. The dose was chosen according to the TSH levels and body weight at the beginning of the study. To assess the equalization of thyroid function in this group of patients, the level of TSH was used as the most sensitive test reflecting thyroid metabolism [27]. Hypothyroidism was considered compensated in patients with TSH levels within the accepted reference standards, i.e., between 0.27 mIU/L and 4.2 mIU/L, along with the absence of symptoms of hypothyroidism. The control group included PCOS patients with normal TSH and FT4 levels, no history of thyroid disease, and not using thyroid hormones or iodine preparations. Due to the lack of TSH and FT4 reference values for the Polish population, the reference standards of the laboratory of the Medical University of Silesia in Katowice were adopted. The adopted range corresponds to the published values of the reference ranges of other Caucasian populations [25].

### 2.3. Measurements

Height and weight were measured in each patient. BMI was calculated as weight (kg) divided by the square of height (m^2^). Blood samples were drawn to quantify thyroid (TSH, FT4) and metabolic (glucose, insulin, total cholesterol, HDL, LDL, triglycerides) parameters. Blood samples were taken after overnight fasting, between the second and fifth day of the menstrual cycle. A fasting 75 g oral glucose tolerance test was performed. Glucose and insulin levels were measured from the samples. The assessment of insulin resistance was carried out using the indirect method using the HOMA-IR index calculated from the formula:

HOMA-IR = fasting serum insulin concentration (*µ*IU/ml) *x* fasting serum glucose concentration (mmol/L)/22.5). Insulin resistance was diagnosed with HOMA–IR values > 2.

In addition, all examined women had routine hormone determinations to conduct the differential diagnosis of PCOS, during which the levels of total testosterone, free testosterone, sex hormone binding globulin (SHBG), follicle-stimulating hormone (FSH), luteinizing hormone (LH), prolactin, 17-hydroxyprogesterone, androstenedione, dehydroepiandrosterone sulfate and cortisol were determined.

### 2.4. Biochemical Analyses

Serum concentrations of TSH, FT4, total testosterone, SHBG, FSH, LH, prolactin, dehydroepiandrosterone sulfate, and cortisol were determined by electrochemiluminescence “ECLIA” using Roche reagents on a Cobas 601 analyzer (Roche Diagnostics GmbH, Mannheim, Germany). Serum glucose, total cholesterol, HDL and LDL cholesterol, and triglycerides were determined by colorimetry (AU 640 analyzer) using Beckman Coulter reagents (Brea, California, USA). Insulin concentrations were determined using the chemiluminescence method (CMIA) using Abbott reagents (Alinity instrument; Chicago, Illinois, USA). The concentration of 17-hydroxyprogesterone and free testosterone was determined using the enzyme immunoassay ELISA from NovaTec (NovaTec Immunodiagnostica GmbH, Dietzenbach, Germany) on a Virclia analyzer (Diamedica, Riga, Latvia). The concentration of androstenedione was determined with the immunochemical test using the chemiluminescence method “CLIA” using Siemens reagents (Immulite 2000 XPi apparatus; Siemens Healthcare GmbH, Erlangen, Germany).

### 2.5. Statistical Analysis

The continuous data are presented as the mean with standard deviation. The normality of the distributions was assessed with the Shapiro Wilk test. Variance homogeneity was assessed by Levene's test. To normalize the values of the selected parameters, the logarithmic transformation was used. In the analysis of the relationships between the studied variables, the Spearman nonparametric correlation test was used. The relationships between the variables are presented in the form of multivariate regression models. The results for which *p* < 0.05 were considered statistically significant. Statistical analysis was performed with the use of SAS 9.2 (Institute INC., Cary, NC, USA) and STATISTICA 12.0 PL (StatSoft Polska, Kraków, Poland).

## 3. Results

The group of women with PCOS and treated SCH had a BMI value significantly higher than the control group. The group of women with untreated SCH did not differ significantly in terms of BMI between the group of treated women and the control group. The analysis of anthropometric parameters, thyroid function, and metabolic parameters of the examined women, taking into account the division into groups, is presented in the table (Table 1.). The mean TSH value in the group of untreated women, 5.58 ± 1.55 mIU/L, differed statistically significantly from the mean TSH in the treated women-2.09 ± 0.92 mIU/L and in the control group-1.62 ± 0.54 mIU/*L* (*p* < 0.0001). FT4 values in all study groups were within the reference standards. There was no statistically significant difference between the groups in any of the analyzed parameters of the lipid profile and carbohydrate metabolism.

### 3.1. Comparison of Metabolic Parameters in Accordance with Nutritional Status

**Figure 1:**
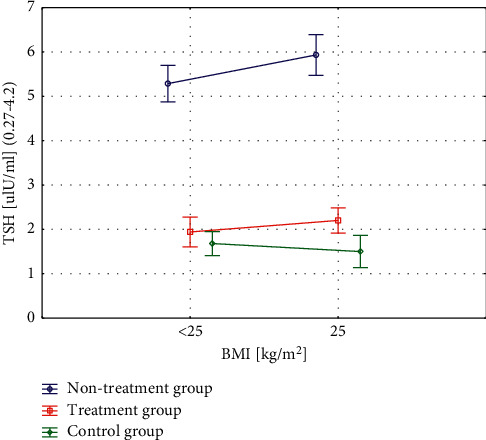
Differences in TSH values related to the nutritional status within the studied subgroups.

Each of the 3 groups was divided into two subgroups: patients with normal BMI (BMI <25) and patients with higher BMI (BMI ≥25) (Table 2). There were no differences in TSH values depending on the nutritional status within the studied subgroups. Significant statistical differences in TSH values were found between the untreated group and the treated and control groups (*p* < 0.0001) (Figure 1).

**Figure 2:**
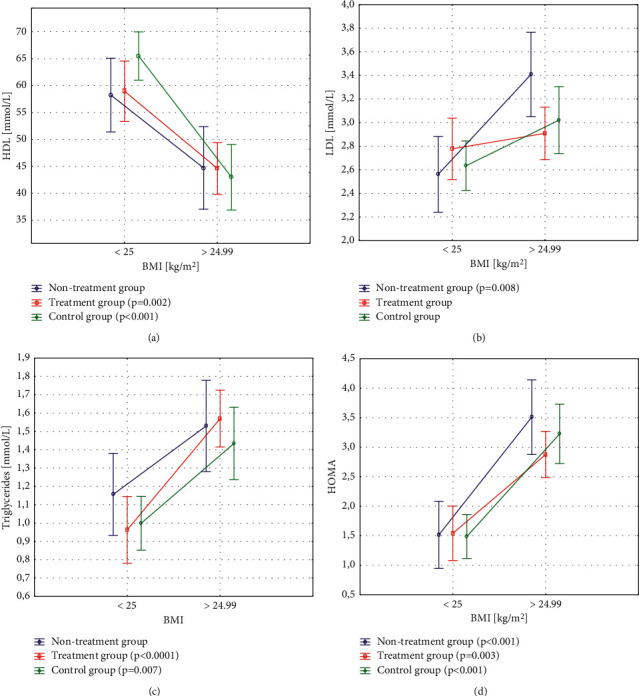
Differences in HDL and LDL cholesterol, triglycerides, and HOMA-IR related to nutritional status within the studied subgroups. (a) In the treated group, HDL cholesterol was significantly higher in women with normal BMI compared to women with higher BMI. (b) LDL cholesterol was higher in the untreated group of overweight and obese woman compared to normal body weight (c) In the group of treated patients and in the control group, triglycerides were significantly higher in patients with higher BMI compared to women with normal BMI. (d) In all study groups, a higher HOMA-IR index was found in the group of overweight and obese women compared to the group with normal body weight. BMI–body mass index; HDL–high-density lipoprotein cholesterol; LDL - light-density lipoprotein cholesterol; HOMA–homeostatic assessment of insulin resistance.

In the study groups: untreated, treated, and control, statistically significant differences were found between patients with normal and higher BMI in terms of lipidogram components: HDL and LDL cholesterol fractions and triglycerides (Figures 2(a)–2(c)). In all study subgroups, statistically significant differences were found between the subgroups in the level of HOMA-IR. Regardless of whether the women belonged to the untreated, treated, or control group, the factor grouping the patients in terms of carbohydrate metabolism parameters turned out to be BMI (Figure 2(d)).

### 3.2. Regression Analysis

In univariate regression models including TSH and BMI as independent variables explaining the dependent variable the value of total cholesterol, it was shown that the variable influencing total cholesterol is TSH. On the other hand, the HDL cholesterol fraction is significantly influenced by BMI, while the LDL fraction and triglycerides are influenced by both TSH and BMI. Therefore, a multiple regression analysis was performed assessing the interaction between TSH and BMI. LDL cholesterol fraction is significantly influenced by TSH, and triglycerides are significantly influenced by BMI. For the remaining variables, such as: glucose, insulin, HOMA-IR, the dominant influence of BMI was shown. Moreover, there was no evidence of collinearity between TSH and BMI (*R* = 0.05; *p*=0.04).

## 4. Discussion

The results of the analysis of the lipid profile in the studied groups are coincident with the results of the work of the group Benetti-Pinto et al. [6], Ganie et al. [19], Pergialiotis et al. [22] and the two meta-analyses [23, 28]. The first group of researchers showed that SCH accompanying PCOS predisposes to higher levels of LDL cholesterol fraction [6]. Ganie et al. [19] showed higher triglyceride levels in the studied group. In the conclusions of the meta-analysis by Pergialotis et al. [22], it was found that SCH causes a clinically mild deviation in the lipid profile in PCOS: lower HDL levels and higher triglyceride levels. Moreover, Huang et al. [29] found no differences in the lipid profile of PCOS and SCH patients between the four major PCOS phenotypes. They found a significant correlation between the level of TSH and the LDL cholesterol fraction, and then determined the TSH cut-off point of 4.07 mIU/L, which is a risk factor for elevated LDL cholesterol levels [29].

There are many possible causes of discrepancy in the results of lipid profile studies in SCH. These include age, race, sex, degree, and duration of hypothyroidism. Moreover, in the conducted studies, the occurrence of insulin resistance is often not taken into account-insulin is a hormone influencing 3-hydroxy-3-methylglutaryl-CoA reductase (HMG-CoA) regardless of thyroid hormones [30] and nicotinism, which are considered modulators of thyroid hormone action on lipid metabolism [31]. Total cholesterol and LDL fraction levels are around 25% higher in smokers with hypothyroidism compared to non-smokers [31].

Final univariate and multivariate regression analysis showed that BMI, not TSH, is a factor that significantly influences glucose levels and the HOMA-IR index. In 2009, Mueller et al. [18, 32] described a significant dependence between the TSH value and insulin resistance in women with PCOS, regardless of the age and BMI of patients. They considered TSH ≥2 mIU/L as the cut-off point characterized by the best sensitivity and specificity for the identification of women with PCOS and insulin resistance [32]. The mean values of the insulin resistance index HOMA-IR in the groups with TSH <2 mIU/L and ≥2 mIU/L were 1.61 and 2.56, respectively [18]. In 2012, subsequent researchers presented results in which they found a statistically significant higher HOMA-IR index in the group of PCOS and SH patients compared to PCOS patients [20, 33]. The limitation of these studies, however, is the small number and statistically significantly higher BMI in the group of patients with hypothyroidism. Subsequent reports did not confirm a significant influence of SCH on the parameters of carbohydrate metabolism [6, 21–23], which is consistent with the results of the presented analysis. The previously cited meta-analyses of studies in this area summarized that SH may have a slight effect on insulin resistance in PCOS, but due to the insufficient number of variables describing carbohydrate metabolism in some of the analyzed studies, unambiguous conclusions in this regard cannot be drawn [22, 23].

In the obtained results, attention were drawn to the significantly higher BMI in the group of patients with compensated hypothyroidism under levothyroxine treatment compared to the control group (27.12 ± 5.59 vs. 25.05 ± 6.44; *p*=0.01). A similar relationship was found by Trummer et al. [34]. The obtained results are consistent with the thesis that the control of thyroid function in SCH does not seem to have a significant impact on the BMI of patients [35]. Although a prospective randomized study with a control group showed a significant weight loss of 0.6 kg after 3 months, it should be noted that patients with the initial mean TSH level of about 5.4 mIU/L received levothyroxine at a dose of 100* μ*g/day, as a result, achieving TSH in the lower range of normal of the reference −0.5 mIU/L [36].

Analyzing the lipid profile in the group of patients with compensated hypothyroidism, the lowest HDL cholesterol level among the studied groups was found. The remaining components of the lipid profile: total cholesterol, LDL cholesterol fraction, and triglycerides were higher than in the control group, but lower than in the untreated group. However, the observed differences did not reach the level of statistical significance.

The results obtained in the group of patients with PCOS and compensated hypothyroidism confirm the existence of a tendency for an unfavorable effect of SCH on lipid parameters in PCOS, although a higher BMI is an additional important factor modulating the results in this group. In the mentioned study by Trummer et al. [34], a lower HDL cholesterol fraction level was also noted when compared to the group of PCOS patients without hypothyroidism. This relationship was statistically significant. The patients did not differ significantly in the remaining parameters of the lipid profile.

Thus far, twelve randomized studies have been conducted to assess the effect of levothyroxine treatment on the lipid profile in SCH. A meta-analysis by Li et al. [37] showed that the level of total cholesterol decreased by an average of 0.29 mmol/L, LDL fraction by an average of 0.22 mmol/L, while the concentration of HDL cholesterol fraction and triglycerides remained unchanged. The reduction effect was weaker, but still statistically significant, in the case of longer treatment for over 6 months, as well as in the treatment of patients with mild hypothyroidism with initial TSH <10 mIU/L [37].

In none of the analyzed parameters of carbohydrate metabolism, there was a statistically significant difference between the group of patients with PCOS and treated hypothyroidism, and the other groups. Similar results were obtained by the authors analyzing patients with PCOS and compensated hypothyroidism [34]. Studies conducted among patients with SCH confirm the lack of impact of levothyroxine treatment on the results of the glucose tolerance test and insulin resistance measured by indirect methods, such as HOMA-IR or quantitative insulin sensitivity check index (QUICKI) [38]. However, the evaluation using the metabolic euglycemic clamp reveals a subtle effect of the treatment of hypothyroidism on the increase in insulin sensitivity [35]. These reports confirm the effect of hypothyroidism on the parameters of carbohydrate metabolism, which, however, is not of great clinical significance in the case of mild hypothyroidism.

The division of the three main study groups into subgroups with normal and increased BMI revealed that the nutritional status is a factor that groups patients in terms of the values of the studied metabolic parameters. The values of the components in the lipidogram in all groups with normal BMI were within the normal range, while in patients with untreated hypothyroidism and BMI ≥ 25, total cholesterol and LDL cholesterol fraction levels exceeded the upper limit of norm. Similarly, the analysis of the distribution of carbohydrate metabolism parameters showed significant differences between the values of insulin and HOMA-IR depending on the nutritional status. Insulin resistance was found in all groups with increased BMI. The highest HOMA-IR index of 3.51 was reported in patients with PCOS and untreated hypothyroidism, and the lowest in patients with PCOS and hypothyroidism during treatment −2.88.

The presented results show that the influence of SCH on metabolic and hormonal parameters in PCOS is modulated by BMI. Subtle thyroid hormone deficiency has no significant clinical effects in lean women with PCOS, but in the case of overweight or obesity, even a slight hormone deficiency, superimposed on existing disorders, can produce clinical symptoms. This is consistent with the results of the study, which analyzed metabolic and hormonal parameters in two groups of PCOS and SCH patients: with BMI <27 and BMI ≥27 [8]. Importantly, in the study by Tagliaferri et al. [8], the carbohydrate metabolism was assessed using the euglycemic metabolic clamp method. Significant correlations between TSH and insulin sensitivity, insulin secretion, and SHBG were found only in the group with BMI ≥27. The multivariate regression analysis, similarly to our study, showed a significant influence of BMI on the components of the lipid profile, fasting insulin and glucose levels, insulin sensitivity, and insulin secretion.

### 4.1. Strengths and Limitations of the Study

The presented work is the second scientific report in which an assessment of the metabolic and hormonal profile of patients with PCOS and SCH under levothyroxine treatment was undertaken. There have been no prospective studies assessing the effects of levothyroxine treatment in this group of patients.

The strength of the presented study is the selection of the study groups-the patients were homogeneous in terms of age, race, degree of hypothyroidism, and insulin resistance. Only patients with a full-blown PCOS–phenotype A were enrolled in the study, which was not specified in many reports so far. In the presented study, the group of women with PCOS and untreated hypothyroidism did not differ significantly in terms of BMI compared to women with PCOS and treated hypothyroidism and women with PCOS without hypothyroidism (26.17 ± 7.01 vs. 27.12 ± 5.59 vs. 25.05 ± 6.44), which made it possible to exclude the modulation of insulin resistance in the studied groups by BMI.

One of the limitations of the study is the recruitment of patients for the study during diagnostic hospitalization in a hospital setting, which may cause overrepresentation of patients with more severe symptoms of hormonal disorders.

In addition, the assessment of insulin resistance was carried out using the indirect mathematical HOMA-IR model, and not the “gold standard” direct method of the euglycemic metabolic clamp. It is a method that is less invasive for the patient, less time-consuming, and does not require the use of appropriate equipment.

The study did not verify cigarette smoking by the patients, or the duration of hypothyroidism in the group of patients treated with levothyroxine, which could affect the parameters of thyroid function and metabolic parameters in the study group. It should be noted that the effects of treating hypothyroidism with levothyroxine in PCOS patients were not prospectively analyzed, which would enrich the cognitive value of the work.

## 5. Conclusions

SCH causes a clinically mild deviation in the lipid profile in PCOS: higher levels of total cholesterol and LDL cholesterol fraction. However, the treatment of SCH accompanying PCOS does not significantly improve lipid and glucose metabolism. It seems that SCH is not able to significantly aggravate the already existing disorders of carbohydrate metabolism and affect the lipid profile of women with full-blown PCOS. An interesting and important direction (due to the frequency of occurrence of the mentioned endocrinopathies) of future research would be to explain the mechanisms of metabolic disorders in the case of coexistence of both endocrinopathies.

The influence of subclinical hypothyroidism on metabolic parameters in PCOS is modulated by BMI. Subtle thyroid hormone deficiency has no significant clinical effects in lean women with PCOS, but in the case of obesity, even a slight hormone deficiency, superimposed on existing disorders, can produce clinical symptoms. This is an argument that justifies the regular control of the parameters of thyroid function in the group of PCOS patients, and in a conversation with an obese patient, it allows to emphasize the role of weight reduction in the case of accompanying hypothyroidism.

## Figures and Tables

**Table 1:** Anthropometric, thyroid, and metabolic characteristics in PCOS women with untreated SCH (non-treatment), treated SCH (treatment), and normal thyroid function (control).

Variable	Group			*p* Value
	Non-treatment *n* = 38	Treatment *n* = 76	Control *n* = 76	
Age (years)	24.21 ± 4.19	25.24 ± 4.29	24.80 ± 3.92	0.4
BMI (kg/m2)	26.17 ± 7.01	27.12 ± 5.59	25.05 ± 6.44	0.01
TSH (mIU/L) (0.27–4.2)	5.58 ± 1.55	2.09 ± 0.92	1.62 ± 0.54	<0.0001
FT4 (pmol/L) (9.01–21.88)	13.42 ± 2.12	15.85 ± 2.58	13.69 ± 1.70	<0.001
CHOL (mmol/l) (<5.2)	4.89 ± 0.92	4.77 ± 0.9	4.72 ± 0.75	0.7
HDL-C (mmol/l) (>1)	1.35 ± 0.38	1.32 ± 0.33	1.46 ± 0.42	0.1
LDL-C (mmol/l) (<3.4)	2.94 ± 0.9	2.85 ± 0.76	2.77 ± 0.72	0.7
TRIG (mmol/l) (<1.7)	1.34 ± 0.58	1.33 ± 0.64	1.17 ± 0.5	0.3
Glucose (mmol/l) (4.08–5.84)	4.79 ± 0.35	4.88 ± 0.39	4.83 ± 0.62	0.5
Insulin [uIU/ml] (<24)	11.08 ± 7.26	10.40 ± 6.00	9.44 ± 6.12	0.2
HOMA-IR	2.41 ± 1.67	2.31 ± 1.44	2.10 ± 1.57	0.2

BMI, body mass index; FT4 – free thyroxine; CHOL, total cholesterol; HDL-C, high-density lipoprotein cholesterol; LDL-C, light-density lipoprotein cholesterol; TRIG, triglycerides; HOMA-IR, homeostatic assessment of insulin resistance.

**Table 2:** Anthropometric, thyroid, and metabolic characteristics in PCOS women in accordance with nutritional status. Each of the 3 groups untreated SCH, treated SCH, and control group was divided into two subgroups: patients with normal BMI (BMI <25) and patients with overweight/obesity (BMI ≥25).

Variable	Group					
	Non-treatment		Treatment		Control	
	Normal BMI *n* = 21	BMI ≥25 *n* = 17	Normal BMI *n* = 32	BMI ≥25 *n* = 44	Normal BMI *n* = 49	BMI ≥25 *n* = 27
Age (years)	24.57 ± 3.93	23.76 ± 4.56	25.06 ± 4.20	25.36 ± 4.39	24.53 ± 3.48	25.30 ± 4.63
BMI (kg/m2)	21.25 ± 2.07	32.25 ± 6.08	21.51 ± 2.15	31.20 ± 3.28	21.18 ± 1.71	32.07 ± 5.91
TSH (mIU/L) (0.27–4.2)	5.29 ± 1.23	5.93 ± 1.85	1.94 ± 0.89	2.20 ± 0.94	1.68 ± 0.52	1.50 ± 0.90
CHOL (mmol/l) (<5.2)	4.56 ± 0.67	5.3 ± 1.04	4.71 ± 0.97	4.82 ± 0.86	4.71 ± 0.77	4.74 ± 1.06
HDL-C (mmol/l) (>1)	1.48 ± 0.42	1.2 ± 0.26	1.49 ± 0.3	1.19 ± 0.29	1.63 ± 0.39	1.63 ± 0.39
LDL-C (mmol/l) (<3.4)	2.56 ± 0.49	3.4 ± 1.08	2.77 ± 0.76	2.9 ± 0.76	2.63 ± 0.64	3.02 ± 0.85
TRIG (mmol/l) (<1.7)	1.17 ± 0.6	1.5 ± 0.51	0.97 ± 0.45	1.59 ± 0.64	1.01 ± 0.4	1.45 ± 1.06
Glucose (mmol/l) (4.08–5.84)	4.7 ± 0.27	4.9 ± 0.41	4.71 ± 0.38	5.0 ± 0.36	4.69 ± 0.57	5.08 ± 0.88
Insulin [uIU/ml] (<24)	7.19 ± 3.38	15.89 ± 7.94	7.25 ± 3.55	12.70 ± 6.40	7.02 ± 2.59	13.84 ± 11.45
HOMA-IR	1.52 ± 0.71	3.51 ± 1.87	1.54 ± 0.80	2.88 ± 1.55	1.49 ± 0.61	3.23 ± 2.63

BMI, body mass index; CHOL, total cholesterol; HDL-C, high-density lipoprotein cholesterol; LDL-C, light-density lipoprotein cholesterol; TRIG, triglycerides; HOMA-IR, homeostatic assessment of insulin resistance.

## Data Availability

The data that support the findings of this study are available from the corresponding author upon request.
